# Parent–Child Discrepancy on Children’s Body Weight Perception: The Role of Attachment Security

**DOI:** 10.3389/fpsyg.2017.01500

**Published:** 2017-09-07

**Authors:** Arcangelo Uccula, Gianfranco Nuvoli

**Affiliations:** Department of History, Human Sciences and Education, University of Sassari Sassari, Italy

**Keywords:** weight perception, parental perception, child weight, attachment security, parent–child agreement, parental attribution, parent–child relations, attachment behavior

## Abstract

The discrepancies between parents and their children on the description of the behavior and representations of their children have been shown in various studies. Other researchers have reported the parents’ difficulty in correctly identifying the weight status of their children. The purpose of our study was to investigate the parent’s attributional accuracy on their children’s body weight perception in relation to the children attachment security. It was hypothesized that insecure children’s parents have a greater discrepancy with their children compared to secure children with their parents. The research participants were 217 children, aged between 5 and 11 years of both genders, and their parents. The attachment pattern was measured by the SAT of Klagsbrun and Bowlby, with the Italian version of Attili. The children were also shown a set of figure body-drawings with which to measure the perception of their weight status. Parents answered a questionnaire to find out the parental attribution of their children’s perception. The results show that the body weight perception of insecure children’s parents have a greater discrepancy with their children’s body weight perception compared with parentally secure children. In particular, parents of insecure children tend to underestimate the perception of their children. This result is most evident in disorganized children. In addition, the perception of insecure children’s parents show a greater correlation with children’s actual weight rather than with their children’s perception. These results suggest that the discrepancies on the perception of children’s body weight between parents and children may be influenced by the poor parental attunement to their children’s internal states, which characterizes the insecure parent–child attachment relationship.

## Introduction

Many studies have highlighted the difficulty of parents in recognizing the body weight of their children (Tompkins et al., 2015). The social significance of this problem is associated with the growing rise in obesity among children in Western countries. Overweight and obesity result from a continuing imbalance between energy intake and energy expenditure, which causes an increase of the body mass index (BMI) due to excessive food intake. These conditions are influenced by the gene–environment interaction in which the environmental component includes sedentary life-style, socioeconomic status, and parenting and feedings styles. Indulgent parenting and specific feeding practices, such as restrictive ones, are associated with the risk of overweight and obesity. Often the adoption of these parental practices is a response to the behavior of the children, but the parents’ perception of their children’s weight may also influence the use of specific feeding styles or practices (Shloim et al., 2015).

A recent meta-analysis shows that the majority of parents do not recognize that their children are overweight (Rietmeijer-Mentink et al., 2013; Tompkins et al., 2015).

Despite this growing body of evidence, there is no clear explanation for the parental misperception of their children’s weight (Towns and D’Auria, 2009). Many researchers reported in different countries a difference between actual and perceived weight in children and adolescents, and this is more common among overweight and obese subjects.

In addition, the method of cross-informant agreement for describing the behavior of children has also highlighted various problems. The correspondence between various informants has been investigated in several studies (i.e., whether parents and children agree on their reports) (Achenbach, 2011) in particular in the clinical setting. A point of convergence has been found between the examined studies concerning the lack of agreement among observers about the symptoms of children and adolescents. The parental–child agreement disclosed on internalizing and externalizing problems is low or moderate (Achenbach et al., 1987; Stanger and Lewis, 1993). Low levels of correspondence between informants often create a great deal of uncertainty and pose a major challenge for the interpretation of research findings on child development (Hourigan et al., 2011). However, these discrepancies may provide a unique opportunity for researchers to better understand the underlying reasons for the lack of agreement (i.e., parental functioning, parental–child interaction and the family dynamics) (De Los Reyes and Kazdin, 2006; De Los Reyes, 2011).

Lagattuta et al. (2012) assessed the direction of the correspondence of the informants in children aged from 4 to 11 years. They found a *positivity bias* in parents with respect to children’s self-reports. These results are in line with previous researches in other developmental domains (e.g., Youngstrom et al., 1999). It is crucial to understand the factors that could explain these low correlations in order to find a way to reconcile the conflict of information, which is coming from different sources, to improve the accuracy of the information and to understand the onset and manifestation of weight problems in children.

The attachment theory provides a starting point from which to explain the discrepancies in the reports (e.g., Berger et al., 2005). According to the attachment theory, a child who experiences constant care and attention from the caregiver develops a secure attachment organization. Individuals with secure internal working models have confidence that their attachment figures will provide a safe haven and a safe support when the child feels in difficulty. As a result, these children learn that it is useful to seek support and to express their discomfort openly to their caregiver, without either exaggerating or minimizing its intensity (Bretherton, 1987).

In the attachment model, mentalization, or reflective functioning, refers to the caregiver’s ability to reflect on the child’s mental experiences to understand and interpret the child’s behavior (Sharp and Fonagy, 2008), in terms of mental states, intentions, feelings, thoughts, motives, and beliefs of her child and herself (Slade, 2005). The mothers’ ability to accurately read the mental states of their infants at 6 months has shown to predict the security of attachment at 12 months (Meins et al., 2001). On the contrary, poor maternal reflective functioning is associated with insecure attachment (Fonagy et al., 1991). Therefore, maternal reflective functioning has been identified by various studies as an important factor of caregiver behavior and parent–child relationship quality, which in turn affects the security of attachment (Belsky and Pasco Fearon, 2008).

These findings are consistent with the hypothesis that the children’s skill in communicating their emotional experiences and distress are associated with having learned over time to have trust in the attachment figure because she/he proved to be responsive and sensitive in providing support.

Also for this reason, it is assumed that the parents of secure children are more tuned to the distress of their children. The mother’s awareness of the self-perception of her child has been shown to be a predictor of security (Allen et al., 2003) and thus an indicator of a lower degree of discrepancy between the child’s self-perception and the perception that others have of him (Vierhaus et al., 2016).

According to the construct of “coherence of mind” (Main et al., 2002, Unpublished), in presence of secure attachment between parents and children, there is a wider agreement due to the increased freedom of communication in their relationship (Ehrlich et al., 2011). This ability is considered a central component of attachment security. However, insecure adolescents, in comparison with secure adolescents, showed a greater discrepancy with their parents regarding the description of their symptoms (Berger et al., 2005). The insecure individuals do not seem able to – or do not want to – discuss coherently their own emotionally painful topics or those related to their psychological symptoms, because they have not had such experiences with their caregivers (Oppenheim et al., 2007).

One’s own weight and body image may also be difficult topics to talk about, because of the central role which they play in the construction of personal identity and self-esteem during development. Divergences from the ideal image or distortions of this central component of the self may be a problem for many children (Evans et al., 2013). In our study, we therefore intend to investigate the parents’ ability to understand how their children perceive themselves regarding their own body weight. In the context of the attachment theory, various researchers have identified the security as a good predictor of parental ability to read their children’s internal states. However, to our knowledge, no study has so far investigated the parental ability to identify the perception that children have of their own body weight in association with the attachment security.

In the present study, we aimed to investigate the parents’ attribution about their children’s perception of their weight status. It is assumed that insecure children’s parents have a greater discrepancy in comparison with the parents of secure children regarding their offspring’s body weight perception. In addition, it is assumed that the parents of insecure children are more affected by the actual body weight of their child rather than by their child’s weight self-perception, because they seem to be less able to read their children’s internal states. The further aim of the research was to explore the direction of the discrepancy in order to investigate whether the parents of insecure children underestimate or overestimate the perception that their children have of their own body weight, in relation to the parents of secure children.

## Materials and Methods

### Participants

This study was conducted by recruiting a sample of children in Northern Sardinia (Italy). Children were recruited from local schools, and were considered as eligible to participate if they had not been diagnosed with a pervasive developmental disorder. In total 217 children (108 females and 109 males) between the ages of 5 and 11, and their parents participated in this study. Average child age was 7.7, respectively (7.82 for boys and 7.69 for girls). In total, 94% of the parent informants were the mothers of the children. The participants were in prevalence from lower middle socioeconomic backgrounds. The proportions distributed across categories of socioeconomic status (SES) according to the occupational qualification were: 24% low, 51.2% mid-low, 12.4% mid-high, and 12.4% high class.

Ethical approval was not required for this study in accordance with the national guidelines, but this paper complies with the rules of the ethical code for research and teaching of the Italian Association of Psychology; it is also in accordance with Declaration of Helsinki – Ethical Principles for Medical Research Involving Human Subjects (WMA, 2013).

### Measures

#### Socio-demographic Variables

Self-reported socio-demographic variables included age, gender, and family residence. Socioeconomic level was based on parental occupational status.

#### Body Mass Index (BMI)

Research followed standardized procedures to measure children’s weight and height at school. BMI was calculated based on height and weight measurements, using the formula BMI = kg/m^2^. To assess child weight status, measured height, and weight was translated into gender and age adjusted BMI percentiles using the guidelines set by the WHO (1995). Each child was classified as *underweight, normal*, or *overweight.*

#### Body Weight Perception

The children’s weight perception was assessed using a figural body-drawing assessment methodology for children. The figures used were developed in a previous study (Monaci and Nuvoli, 2002) and consist of five hand-drawn gender-specific figures. These scales were developed for the age of primary school children. The scale is age-specific and appropriate for the age range of our sample, in line with the recommendations for using these tests in children (Yanover and Thompson, 2009).

The figures are horizontally organized in ascending order of adiposity and represent five different levels of male and female body shapes, ranging in size from 1 (very slim figure) to 5 (very large figure). Figures 1, 2 correspond to underweight subjects, Figure 3 represents normal weight subjects and Figures 4, 5 represent overweight subjects.

#### Parental Attributions of Children’s Weight Perception

Parental attribution was assessed using a multiple-choice question: “How do you think your son/daughter perceives his/her body weight?” The question used was developed in a previous research (Monaci and Nuvoli, 2002). The five response options correspond to those shown in the drawings for the children’s version: My son/daughter “thinks he/she is very slim”; “thinks he/she is slim”; “thinks he/she is normal”; “thinks he/she is heavy”; “thinks he/she is very heavy.”

#### Separation Anxiety Test (SAT)

Attachment patterns were assessed by a modified and validated Italian version (Attili, 2001) of the Separation Anxiety Test (SAT) by Klagsbrun and Bowlby (1976). The SAT is a semi-projective test for children designed to assess children’s responses to scenes depicting separations from their parents. It consists of two sets of six vignettes (one for girls and one for boys) depicting scenes of mild or severe separations from parents (three with long-term separation and three with short-term separation).

After each picture is described, the tester asks “How does the child in the picture feel?”, “Why does she/he feel this way?”, “What do you think she/he will do?” and “What is she/he going to feel/do at reunion with parents.” The scoring system used to classify participants is based on 17 emotional categories such as loneliness, sadness, rejection, anger, well-being, escape, anxiety, withdrawn, etc. These categories are then grouped into eight main classes of responses based on attachment theory (Bowlby, 1969, 1973), that is: attachment security, lack of self-esteem, hostility, self-confidence, avoidance, anxiety, anguish, and confusion.

A score (range -2/+2) is assigned for each answer. The sum of these scores is used to assign an overall score for each subject, to classify him/her into one of the four attachment categories, derived from the theory, in terms of *secure* (summary score of +4 or above); *ambivalent* (summary score ranging between +3 and +1); *avoidant* (summary score ranging between 0 and -2) and *disorganized* (final score of -3 or below).

The Italian version has shown satisfactory concurrent validity (*r* = 0.77; *p* < 0.001) with Klagsbrun and Bowlby’s coding system, high inter-rater reliability (Cohen’s *k* = 0.80; *p* < 0.001) and adequate test–retest correlation (*r* = 0.75; *p* < 0.001) (Attili, 2001).

### Procedure

Informed consent was obtained from the school principals and teachers. All children who consented and who obtained written consent from parents were included. Originally, *N* = 249 children and parents were contacted. All children consented and 12.85% of parents did not give their permission to participate in the study.

Children were given packets containing the questionnaire to take home to their parents. The parents answered the questions about the socio-demographic data and on their children’s body weight attributions. The questionnaires were given to parents in a closed envelope that contained instructions requesting a response from only one of the parent figures (in 94% of the cases the mother answered) and with emphasis about not discussing the responses with their children.

The measurements (weight and height) and the tests (SAT and body weight perception) for the children were completed individually for each child in a quiet room of the school. Test administrators were doctoral researchers who were carefully instructed to follow the instructions for procedures of the SAT and the other measures.

Children selected the picture that best represents their body size perception from a set of five figural drawing scale. Responses were collapsed into three categories: *slim* (including very slim), *normal*, and *heavy* (including very heavy). Furthermore, the parents’ responses were grouped in the same categories.

With regard to the SAT, two independent coders classified all transcripts. Inter-rater agreement was 89.4% (Cohen’s Kappa = 0.84, *p* = 0.000). After the two coders completed the study coding, a third coder served as an independent assessor on the 10.6% discordant encodings. This independent assessment resulted in 100% inter-rater agreement.

#### Discrepancy Estimating Calculation

Estimating categories were calculated by subtracting each child category weight perception from the corresponding parent weight category attribution.

The positive discrepancy (overestimated category) indicates that parents reported higher weight category than children reported; conversely, the negative discrepancy (underestimated category) indicates that children reported higher weight category than parents. Parental–child concordance indicates the accurate parent estimation.

#### Statistical Analyses

Associations and correlation between categorical variables (BMI, children self-perception and parental attribution) were assessed using the χ2 and Spearman’s correlation tests. Multivariate logistic regression analyses were performed in order to assess the statistical significance of the associations of attachment security/patterns, gender, age and socioeconomic level (independent variables), with the category of parental accuracy of perception (dependent variable). Odds ratios (OR), their respective 95% confidence intervals (CI) and *p*-values were reported. Results were considered to be statistically significant at *p* ≤ 0.05.

## Results

### Attachment Patterns

Ainsworth et al. (1978) identified the first three styles, one secure and two insecure (ambivalent and avoidant), and subsequently Mary Main identified the fourth style, which was defined disorganized and added to the insecure category (Main and Solomon, 1986).

Following the SAT decoding, in our sample secure attachment is the prevailing style (52.6%); the ambivalent style (17.5%) and the avoidant style (17.5%) have the same percentages; there are also 12.4% of children who show the disorganized style. For some statistical analyses, we used two groups: secure (52.6%) and insecure (47.4%), by combining the ambivalent, avoidant, and disorganized patterns.

### Children’s Weight Categories, Children’s Self-perception and Parental Attribution

**Table 1** displays the frequencies and percentages of the three weight categories according to the children’s measured BMI, the categories of children’s weight self-perception and parental attribution of their child’s weight self-perception. Over half of the sample falls within a normal weight BMI, a third is overweight and approximately 10% is underweight. From the analysis of the children’s self-perception, it emerges that more than double the children who are actually underweight describe themselves as such; the percentage of those who define themselves as normal weight is similar to the corresponding measured BMI; and finally, the children who perceive themselves overweight are far fewer than those who are in actual fact overweight. On the other hand, parents describe their children as underweight to a lesser extent, and as overweight and normal-weight in a slightly greater extent than stated by the children themselves.

**Table 1:** Frequencies and percentages of the children’s BMI, children’s weight perception and parental attribution.

Weight categories	Children’s measured BMI	Children’s self-perception	Parental attribution
Underweight	23 (10.6)	60 (27.6)	45 (20.8)
Normal	122 (56.2)	114 (52.6)	122 (56.2)
Overweight	72 (33.2)	43 (19.8)	50 (23.0)

The analysis of the degree of agreement between parents and their children on children’s weight self-perception reveals a general parental tendency to overestimate children’s leanness self-image and to underestimate the perception that the children have of themselves when they feel fat. There is a parent–child agreement in normal-weight perception in two out of three of all cases (**Table 2**).

**Table 2:** Children’s weight perception and parental attribution.

Children	Parents			
Weight perception categories	Slim	Normal	Heavy	Total
Slim	36.7	48.3	15.0	100
Normal	14.9	63.2	21.9	100
Heavy	14.0	48.8	37.2	100

χ^2^*= 17.71; df = 4; *p* = 0.001*.

### Attachment Security

The distribution of the percentages based on the attachment security are shown in the following tables. The division of the sample in insecure and secure children highlights differences in the percentage distribution. **Table 3** summarizes the percentage of agreement between children and parents on the children’s weight perception. In the group of insecure attachment, agreement rates between parents and children are significantly lower in comparison with the group of secure attachment.

**Table 3:** Children’s weight perception and parental attribution in relation to attachment security.

Children		Parents			
Children security categories	Weight perception categories	Slim	Normal	Heavy	Total
	Slim	29.0	58.1	12.9	100
**Insecure^∗^**	Normal	23.5	56.9	19.6	100
	Heavy	19.0	52.4	28.6	100
					
	Slim	44.9	37.9	17.2	100
**Secure**^∗∗^	Normal	7.9	68.3	23.8	100
	Heavy	9.0	45.5	45.5	100

^∗^χ^2^*= 2.19; df = 4; *p* = 0.699. ^∗∗^χ^*2*^ = 24.44; df = 4; *p* = 0.000*.

In the group of secure children attachment, there is a significant differentiation as two out of three normal weight boys agree with parental perceptions about them, and nearly one out of two agree in the slim and the heavy categories.

**Table 4** describes the Spearman correlations between children’s categories of weight self-perception, parental attribution of their children’s weight perception and BMI categories in relation to attachment categories. The results show that the secure children’s parents showed a significant correlation with their children, while the parents of insecure children show a low correlation. It should be noted that insecure children’s parents have a higher correlation with the actual weight rather than the weight perception of their children.

**Table 4:** Correlation matrix between children’s weight perception, parental attribution and BMI in the attachment security categories.

	Children security categories	BMI categories	Parents
	Insecure	0.146	0.135
**Children**	Secure	0.362^∗∗∗^	0.330^∗∗∗^
	Total	0.253^∗∗∗^	0.239^∗∗∗^
			
	Insecure	0.441^∗∗∗^	–
**Parents**	Secure	0.394^∗∗∗^	–
	Total	0.421^∗∗∗^	–

*^∗∗∗^P_*s*_= 0.000.*

**Table 5** describes the discrepancy direction of parental estimation on their children in relation with insecure and secure attachment. Logistical regression analysis shows that the discrepancy is present in both directions of the underestimation and overestimation, resulting significant especially in the propensity in parents of insecure children to underestimate the weight perception that their children have of themselves (OR = 2.38).

**Table 5:** Category of parental estimation and attachment security.

Estimation category	*N*	Secure^∗^ (%)	Insecure (%)	OR	*p*	IC
Underestimated	44	14.9	26.2	2.38	0.018	1.16–4.88
Accurate^∗^	110	57.9	42.7	1	–	–
Overestimated	63	27.2	31.1	1.55	0.170	0.83–2.89

*^∗^ Reference category; CI indicates confidence Interval; OR, odds ratio*.

**Table 6** describes the percentage distribution of parental accuracy estimate broken down into the four attachment categories. Logistical regression analysis shows that the propensity to discrepancy increases progressively between parents and their children, starting from the category of secure attachment. In the ambivalent and avoidant category a greater discrepancy emerges in comparison to the secure category, which becomes statistically significant in the disorganized style (OR = 2.75).

**Table 6:** Attachment patterns and parental accuracy on children’s weight perception.

Attachment categories	*N*	Accurate^∗^ (%)	Inaccurate (%)	OR	*p*	CI
Disorganized	27	33.3	66.7	2.75	0.025	1.14–6.64
Avoidant	38	44.7	55.3	1.70	0.160	0.81–3.56
Ambivalent	38	47.4	52.6	1.53	0.260	0.73–3.19
Secure^∗^	114	57.9	42.1	1	–	–

*^∗^ Reference category; CI indicates confidence interval; OR, odds ratio*.

## Discussion

Our study fits into the topic of research that investigates the level of discrepancy between parents and their offspring concerning children’s representations and behaviors. Our purpose is to make a contribution, in a non-clinical sample, on the level of discrepancy between parents and their children with regard to the perception of body weight that children have of themselves. The degree of discrepancy has been analyzed, taking into consideration the children’s attachment security.

Our hypothesis is that the level of parent–child discrepancy is associated with the type of attachment security of the child, and thus it is greater in the case of insecure attachment, and inferior in the case of secure attachment.

Our findings suggest that parents of secure children have a greater accuracy in their ability to attribute internal mental states to their children’s weight perception. The insecure children’s parents, on the other hand, express greater discrepancy with the image that their children have of themselves.

The scientific literature that highlights the low parent–child agreement does not provide an exhaustive description of the causes of the discrepancy, and usually does not go beyond the description of the observed phenomenon. The methodology of using different reporters, which measures the degree of discrepancy between different perspectives (Hunsley and Mash, 2007; De Los Reyes et al., 2013), is used to fill the lack of a shared and objective method with which to describe the behavior of the child, especially when clinical syndromes are present (Achenbach, 2006). According to De Los Reyes and Kazdin (2005), these studies lack a theoretical conceptualization, and indeed, the discrepancy found between the different observers is sometimes attributed to the context, or to the characteristics of the participants who provide the information from time to time. Various studies have shown that the qualitative aspects of the parent–child relationship appeared to contribute more strongly to parent–child discrepancies than the socio-demographic factors did (Van Roy et al., 2010). Among the studied aspects, parental engagement and a good communication between parents and their children are important to increase parent–child agreement (Treutler and Epkins, 2003).

The Attachment Theory in this study may provide a contribution in general to the theoretical conceptualization concerning the nature of these differences and in the field of children’s body weight perception in particular. In the attachment theory, the concept of *sensitivity* was fundamental to understanding the underlying factors in the building of security: the mother of a secure child appears to be able to “perceive things from the point of view of children” (Ainsworth et al., 1971). Recent studies deepen this component of the relation by identifying more specific factors, such as the ability of mothers to engage in mind-mindedness and therefore consider the child as a mental agent (Meins, 1997). The concepts of *mind-mindedness* (Meins, 1997, 2013), *mentalizing* (Frith and Frith, 2003), and *reflective functioning* (Fonagy et al., 1991) in relation to the attachment theory may help to explain the increased capacity of the secure children’s parents to properly attribute the internal states of their children, on the contrary to the insecure ones.

In the early stages of development, the parents’ ability to try to make meaning of the child’s mental state at 6 months promotes the development of a secure attachment at 1 year (Meins et al., 2001). In children’s development at later stages, at 7–11 years old, Sharp et al. (2006) have investigated the mothers’ ability to assess their children’s social-life attributional style. They found that low maternal accuracy is associated with children’s low psychosocial adjustment and high degree of psychopathology. In our study, the lack of accuracy with which the parents think their children perceive their body weight is associated with children’s insecure attachment, especially with the disorganized pattern.

According to the attachment theory, affective communication between the caregiver and the disorganized-pattern child is characterized by a low attunement by mothers to the children’s signals, and by a caregiving style that is often based on competition (Lyons-Ruth et al., 1999). The attachment insecurity has also highlighted in our data a low capacity of mothers to accurately perceive the representation that their children have about their own body weight. This result can come from a greater parent–child conflict and is hypothesized in the presence of a discrepancy between the perspectives (De Los Reyes and Kazdin, 2006).

In our study, the perception of insecure children’s parents shows a low correlation with their children’s representation, and on the contrary, they showed a higher correlation with their sons’/daughters’ actual weight. In other studies, parents’ perception of the children’s internal states can be difficult because the parents’ attention is focused on the children’s overt behavior; in particular, when children have a behavioral disorder of the externalizing type such as attention and hyperactivity disorder (Kemper et al., 2003).

In our results, in the insecure child category, the greater correlation between parent attribution and actual weight rather than the perception that children have of their own weight, could suggest a greater influence by the image of children”s actual body size, instead of what their children really think of themselves. This result could provide a further indication of the poor parental attunement to their children’s internal states, which would tend to shift the parents’ focus from their children’s representation to their actual body weight.

The direction of the discrepancy is another finding that emerges from the analysis of our data. Insecure children’s parents express the discrepancy in particular underestimating their offspring’s weight representations. They think that their children perceive themselves leaner than how they actually stated. Various studies have demonstrated the parent’s tendency to underestimate the actual weight of their children. A particularly large proportion of overweight and obese children, as well as their parents, underestimate children’s weight status (Rietmeijer-Mentink et al., 2013).

These studies show a tendency of parents to underestimate their children’s actual weight, in particular when overweight, but the difficulty of parental estimation is also confirmed when compared to the points of view of parents and children on the children’s own features. Researchers who have examined the directional differences between parent and child suggest a parental positivity *bias* (Lagattuta et al., 2012). This tendency to a more positive view occurs especially for socially undesirable symptoms (Comer and Kendall, 2004).

We think that the discrepancy between parent attribution and child’s weight perception, in general, is associated with a poor parental sensitive attunement to infant’s internal states when the child attachment is insecure. The parental underestimation of their children’s weight perception that emerges in insecure family contexts can be attributed to the greater influence of social desirability that leads parents to think that their children feel leaner than the parents perceive them.

### Limits, Implications for Research and Practice

Our aim has been to analyze the discrepancy of child weight between the two partners in relation to the attachment security of the child. Our study was not designed to provide information on the ability of parents to properly perceive the body weight of their children, a goal that has been the subject of other investigations (Rietmeijer-Mentink et al., 2013; Tompkins et al., 2015).

The mother–child perspective differences were usually examined in the clinical evaluation contexts, in which it is necessary to have more perspectives of the behavior of the child where the discrepancies can be conceptualized as a component of the type of family relations. In similar contexts, the reporters may have different motives in providing the information, or different thresholds in the evaluation of what could be, for example, an atypical behavior (Richters, 1992). In the parents’ evaluation of their children’s body weight, this aspect can be important because of the conventions that may be present in a family about the meaning of the concepts of *slim*, *normal*, or *heavy*. The mothers, in fact, can tend to consider their maternal competency based on how much their child eats (Chatoor et al., 2000).

We examined parent–child reporting discrepancies using different assessment methods: a questionnaire versus a set of drawings. Although the structure of the two scales was similar (five drawings and five questions describing a visual and a verbal mode of the same sequence of body sizes), this difference of used instruments can contribute to the discrepancies. Nevertheless, different assessments tools reported by different informants are often used in research (Kraemer et al., 2003), therefore arguably the findings of the present study are ecologically valid; in addition, for children the use of drawings seems more appropriate and for adults, the questionnaire is more appropriate.

In addition, the cultural influence and the history of the mother’s relationship with her own body image may affect her assessment of the body weight of her children. De Los Reyes and Kazdin, (2006) suggest that it would be appropriate to study the discrepancy as a central factor for better understanding the dyadic interaction and the functioning of the parents, the child and the family dynamic.

Also, our study was not designed to provide information on the satisfaction on their body image in children. Naturally, even the perception that children have of their body weight can be affected by the degree of satisfaction they have with their body image. Some researchers found that nearly half of children between 6 and 12 are unhappy about their appearance (McCabe and Ricciardelli, 2005). Various studies investigating the body dissatisfaction in children and adults have applied the attachment theory. The research in this area showed that insecure attachment is associated with body dissatisfaction problems in adulthood and adolescence. Moreover, this association was also found in children (Goossens et al., 2012).

A greater concern for the weight seems to be associated with the attachment insecurity. The low self-esteem of children with insecure attachment increases sensitivity to appearance-related messages and makes them more vulnerable to developing problems associated with body image (Sharpe et al., 1998). Cheng and Malinkcrodt (2009) specifically report that attachment anxiety mediates the relationship between parents and children’s body dissatisfaction. The parents, in fact, play an important mediating role between body weight and self-concept (Smolak et al., 1999), in which the maternal concern about body weight of children is associated with a low body esteem in children.

Further studies on the discrepancy between parents and children on the perception of children’s weight could measure both the attachment styles of children and their parents. They could also evaluate how body-image dissatisfaction in children and parents can influence the discrepancy between the two partners (parents and children) on the perception of the child’s body weight. Finally, there are many body weight implications in terms of the children’s self-concept development, both on the role of distortion and dissatisfaction of their image and on the potential evolution in eating disorders. For this reason, programs for prevention and education aimed at increasing the awareness of parents about their role in body weight development of their children have been proposed (McCabe et al., 2014). The success of educational interventions largely depends on the subjective ability to perceive their weight status (Bennett and Wolin, 2006). Accurate weight perception may be a predictor of healthy management (Chung et al., 2013) and inaccurate weight perception may be an obstacle for weight and food management, for the prevention of eating disorders, and for educational programs about what is a healthy weight (Videon and Manning, 2003). Findings from this study suggest the importance of weight perception in eating behavior and in healthy management, in order to promote psychological well-being and reduce the prevalence of overweight–obesity. As also shown in our research, in insecure relationship contexts of attachment in particular, the parents can play an important role in allowing distortions in the development of correct perception of children’s weight and body image.

## Author Contributions

AU: Concept of the study, statistical analysis and data interpretation, writing of the article. GN: Concept of the study, data interpretation, and proof reading.

## Conflict of Interest Statement

The authors declare that the research was conducted in the absence of any commercial or financial relationships that could be construed as a potential conflict of interest.
